# What is the effect of supervised rehabilitation regime vs. self-management instruction following unicompartmental knee arthroplasty? – a pilot study in two cohorts

**DOI:** 10.1186/s40634-021-00354-x

**Published:** 2021-06-09

**Authors:** Adam Omari, Lina Holm Ingelsrud, Thomas Quaade Bandholm, Susanne Irene Lentz, Anders Troelsen, Kirill Gromov

**Affiliations:** 1 University of Copenhagen, Faculty of Health and Medical Sciences, Blegdamsvej 3B, 2200 Copenhagen N, Denmark; 2 Department of Orthopedic Surgery, Copenhagen University Hvidovre Hospital, Kettegård Alle 30, 2650 Hvidovre, Copenhagen, Denmark; 3 Department of Clinical Research, Copenhagen University Hospital, Amager and Hvidovre, Kettegaard Alle 30, 2650 Hvidovre, Copenhagen, Denmark; 4 Department of Physical – and Occupational Therapy, Copenhagen University Hospital, Amager and Hvidovre, Kettegård Alle 30, 2650 Hvidovre, Copenhagen, Denmark

**Keywords:** Unicompartmental knee arthroplasty, Rehabilitation regime, Functional outcome, Physiotherapy, Mobilization, Range of motion

## Abstract

**Purpose:**

The optimal rehabilitation strategy after a unicompartmental knee arthroplasty (UKA) is unclear. This study aims to compare the effect of transitioning from a supervised to a self-management rehabilitation regime by pilot study of patient outcomes subsequent to UKA surgery.

**Methods:**

Fifty consecutive patients scheduled to undergo unilateral UKA surgery at our institution between 22^nd^ February 2016 and 18^th^of January 2017 were prospectively identified via local medical database and included. Performed UKAs were grouped into two cohorts, Supervised Cohort and Self-management Cohort, temporally separated by introduction of new rehabilitation. Self-management Cohort(*n* = 25) received an extensive inpatient rehabilitation regime along with outpatient referral to rehabilitation center. The Self-management Cohort(*n* = 25) were only instructed in use of crutches and free ambulation at own accord. Follow-up (F/U) was 1 year from receiving UKA. A range of outcomes were recorded, and between-cohort differences compared: knee joint range of motion, pain and functional limitations, length of stay (LOS), readmission rate, pain during activity and rest, and knee circumference.

**Results:**

Complete data was obtained for *n* = 45 patients. The mean between-cohort difference in ROM (range of motion) from preoperatively to discharge was 15.4 degrees (CI:5.2,25.8, *p* = *0.004*), favoring the supervised regime, with no difference detected in any outcome at 3- or 12 months F/U. Median LOS was 1 day in both cohorts.

**Conclusion:**

Transition to a simple rehabilitation regime following UKA surgery was associated with decreased ROM at discharge, which was not present at 3-month F/U. We found no other between-cohort differences for any other outcomes at 3- and 12-month F/U including functional limitations, although the study was likely underpowered for these outcomes. We encourage large-scale replication of these findings using randomized designs.

**Level of evidence:**

Therapeutic level II

## Introduction

Unicompartmental knee arthroplasty (UKA) is a common treatment option for patients with unicompartmental osteoarthritis, who have undergone guideline recommended non-surgical treatment [1] and still experience substantial unicondylar femoro-tibial osteoarthritis with preserved cruciate ligaments. The surgical procedure aims to restore function and reduce pain [2], and accounts for 8–10% of all primary knee arthroplasties in the United Kingdom and United States [3]. Improvements in surgical technique and components along with option of minimal invasive approach has in the past decades transformed UKA into an efficient and reliable procedure [4], with documented improvements in patient reported outcome measures (PROM), range of motion (ROM), kinematics and functional recovery when compared to more invasive total knee arthroplasty (TKA) [5–8]. Meta-analysis of RCTs between UKA and TKA reported standard mean difference of -0.19 in PROMs, risk ratio (RR) of 0.27 for major postoperative complications favoring UKA, but RR of 5.95 for revision rate at 5 years favoring TKA [8].

UKA rehabilitation regimes are often similar to TKA regimes possibly because of the larger scientific evidence base for TKA. Rehabilitation regimes or management after TKA is based on the rationale that it enhances post-operative recovery, which is also reflected in the recent guideline for physical therapist management of TKA published on behalf of the American Physical Therapy Association [9]. It has been difficult to show superiority of high-intensity [10, 11] or supervised [12] rehabilitation exercise regimes over less intense or home-based regimes on post-operative recovery outcomes, such as functional performance and muscle strength. So, because it seems difficult to substantially impact post-operative recovery after TKA when different rehabilitation exercise regimes are compared, we have recently asked whether rehabilitation exercise is superior to no rehabilitation exercise after TKA [13, 14]. This question is also relevant in UKA where the surgical trauma is smaller and where few studies have compared different rehabilitation regimes after UKA [15]. Before starting a large-scale trial in UKA, we wanted to address the question by a pilot trial in which we changed regimen. We undertook this pilot study as a first step to challenge our pre-existing belief that rehabilitation exercise enhances recovery after UKA (and plan future randomized trials).

In this study, we aim to compare the effect of a transition from a supervised to a self-management UKA rehabilitation regime on a range of outcomes which includes; knee joint range of motion, pain and functional limitations (Oxford Knee Score and Forgotten Joint Score), length of stay (LOS), readmission rate < 30 d, pain during activity and rest, and knee circumference before and after inpatient training.

## Materials and methods

This observational pilot study assessed in- and outpatient supervised rehabilitation vs. simple self-management instructions following UKA surgery at our institution. Patients were assessed in-person preoperatively, at discharge, at 3-month, and 12-month follow-up (F/U). Type of measurement varied at each timestamp and altogether included ROM, PROMs and LOS, readmission rate, pain during activity and rest, and knee circumference. Neither surgeons, patients nor physiotherapists were blinded with respect to chosen treatment due to the observational nature of this study. This study adheres to STROBE reporting guidelines using the extension for cohort studies to ensure a complete report of the study’s conduct, design, and findings [16].

We prospectively identified 50 consecutive patients scheduled to undergo medial unilateral UKA at Copenhagen University Hospital Hvidovre between 22^nd^ February 2016 and 18^th^ of January 2017, of whom 25 patients (Supervised Cohort) were operated before change of the usual rehabilitation regime, and 25 patients (Self-management Cohort) were operated after the change was implemented. The change was introduced on August 30th, 2016. Patients operated prior to this date were designated a physiotherapist-supervised inpatient rehabilitation regime that included five unique supine knee exercises in bed, followed by nine unique knee standing exercises prior to discharge. Once discharged the Supervised Cohort was admitted to an outpatient course within seven days consisting of six to eight group-based rehabilitation sessions supervised by trained physiotherapists at local municipality center. On the contrary, patients undergoing UKA surgery after change in the usual rehabilitation regime at our institution (August 2016), followed a self-management regime which merely consisted of encouraged ambulation via crutches. Self-management Cohort was not offered any subsequent outpatient rehabilitation after discharge.

All UKA surgeries were performed using the same medial cementless mobile bearing UKA implants using microplasty instrumentation*.* Patients were intended spinal anaesthesia, multi-modal opioid sparing analgesia, high dose preoperative corticosteroids [17], preoperative intravenous tranexamic acid, no drains, early mobilization with full weight bearing, in-hospital only thromboprophylaxis if LOS ≤ 5 days (0 patients). Tourniquets were used for the duration of surgery at 250 mmHg. Postoperative opioid sparing analgesia consisting of Paracetamol 1 g × 4 and COX2 inhibitors 200 mg × 2 daily was administered for a maximum of 7 days. Opioids are used as rescue medication only. In-hospital only thromboprophylaxis is used according to local guidelines. Discharge to own home once they fulfilled functional discharge criteria: independence in personal care, ability to get in and out of bed, ability to get dressed independently, ability to sit and rise from a toilet/chair, mobilization with walker/crutches, and ability to walk in excess of 70 m with crutches. Fulfilment of discharge criteria was judged by either an experienced nurse or physiotherapists employed at our department. All patients in this study were discharged to their own home. In case any problem arose with respect to fulfilling discharge criteria patients were to remain admitted to the hospital and continue designated rehabilitation.

### Rehabilitation regime for supervised cohort

**Fig. 1:**
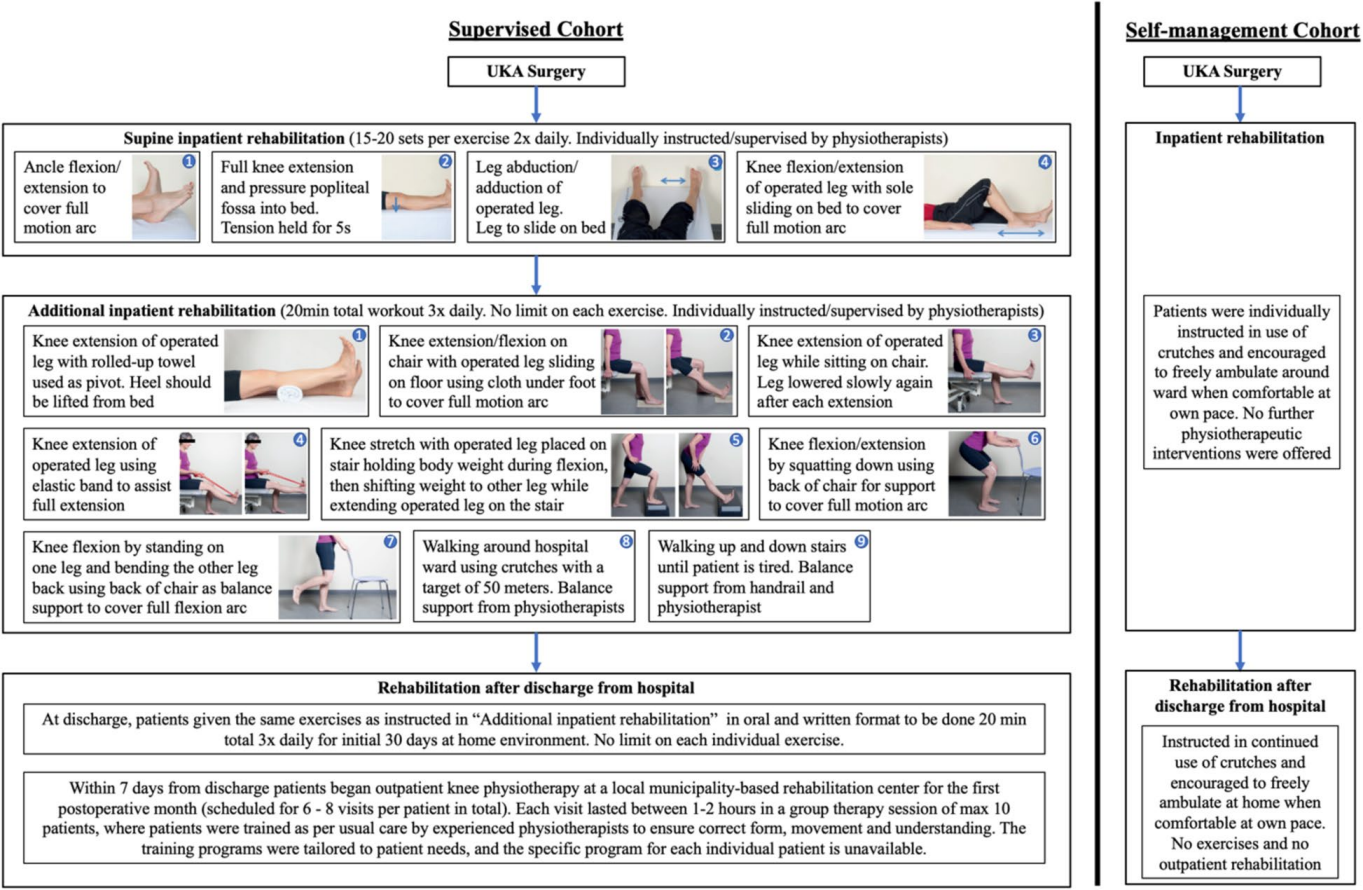
Rehabilitation exercises following UKA surgery for Supervised Cohort and Self-management Cohort

Rehabilitation plan varied greatly depending on designated cohort. Figure 1 outlines a detailed description of the specific rehabilitation exercises in each of the two regimes. Patients in the Supervised Cohort received inpatient rehabilitation through careful instruction by physiotherapists and after discharge outpatient rehabilitation at local rehabilitation center. This inpatient physical rehabilitation entails a predetermined list of specific physical exercises (Fig. 1). Exercises began after operation with the patient still supine in the hospital bed and consisted of knee- and ankle joint flexions and extension along with hip abductions. Each exercise was repeated 15–20 times twice daily starting the day of surgery. Oral encouragement was given to patients by the supervising physiotherapists during exercises to ensure patient satisfaction and resolve any questions or negative emotions. Also, once anesthesia fully wore off and patients were comfortable standing upright and leave their hospital bed, they were then instructed by physiotherapist in nine unique inpatient physical rehabilitation exercises (Fig. 1). This included; walking using crutches, walking up and down a flight of stairs, leg extension/flexion and a range of joint mobility exercises. They were to be done three times daily for a total of 20 min at each separate session (morning, noon and evening), with no limitations on number of repetitions and sets completed in each specific exercise. Supervision by physiotherapists was intended for all sessions in all patients but compliance was not possible to establish. Patients were discharged when they met the described discharge criteria. All patients adhered to this inpatient rehabilitation regime. Adherence to the regime was measured and reported by the supervising physiotherapists in the local medical database while patients were admitted at the hospital.

At discharge, Supervised Cohort received a list of instructions for nine unique exercises in both oral and written format. These were identical to the nine exercises patients performed during inpatient rehabilitation. The instructed exercises were to be done in the exact same format and frequency as prior to discharge in patients own home environment for the first postoperative month. Furthermore, patients in Supervised Cohort simultaneously began outpatient knee physiotherapy within 7 days of discharge at a local municipality-based rehabilitation center during the first postoperative month. A total of six to eight visits per patient were scheduled (approximately twice a week). Variance in specific number of visits was caused by the pragmatic nature of this study. Each outpatient session lasted between one and two hours in a group therapy session of max 10 patients at a time, where patients were trained as per usual care by physiotherapists (Fig. 1). The training programs were tailored to patient needs, and the specific program for each individual patient were unavailable. After the last visit to the outpatient rehabilitation center, patients were exempt from any further rehabilitation measures. Adherence to outpatient physiotherapy could not be measured for Supervised Cohort. This Supervised Cohort rehabilitation regime was identical to the one offered to TKA patients at our institution.

### Rehabilitation regime for self-management cohort

After undergoing UKA surgery, patients in the Self-management Cohort were simply instructed and encouraged in use of crutches at their own accord in the amount the patients themselves deem necessary. Patients were allowed to ambulate freely around the hospital ward at their own pace when comfortable on crutches. Encouragement was given to patients orally by physiotherapists when patients began using crutches to ensure patient satisfaction and resolve any questions or negative emotions. Patients were discharged once they met the described discharge criteria. In case any problem arose with respect to fulfilling discharge criteria patients were to remain admitted to the hospital and continue designated regime until discharge criteria were fulfilled. At discharge, Self-management Cohort received no specific exercises nor admittance to an outpatient rehabilitation track. Instead, they were reassuringly instructed in continued use of crutches and confirmed permission to ambulate freely. Use of crutches after discharge was at the patient’s own accord and in the amount that they found necessary (Fig. 1). Patients could disband crutches once they could walk effortlessly and securely. There were no specific exercise or non-exercise components, modifications or tailored approaches in this self-management rehabilitation regime.

### Outcomes

At preoperative assessment which occurred within three months of surgery, preoperative ROM, knee circumference and Oxford Knee Score (OKS) were measured. Patient demographics were registered comprising of baseline characteristics; age, gender and body mass index (BMI). During surgery, any intraoperative complications were noted by the surgical assistants. Efforts to minimize potential sources of bias were made: the same three experienced orthopeadic surgeons from our institution performed pre-operative assessment and the UKA surgery in all patients to ensure consistency in surgical outcome and pre-operative grading. All patients stayed at our department until discharge. Surgeon and physiotherapist involved in treatment and measurement of patients were naturally aware of change in usual regime and hence not blinded, however, non were aware of the measurements would feature in a scientific study.

Patients were followed postoperatively through use of a unique 10-digit civil registration by crosschecking in the national medical database ensuring minimal loss to F/U and ensuring data on LOS and readmission < 30 days at any Danish hospital. F/U length was 12 months from receiving UKA with in-person recovery assessment conducted after 3- and 12-months. ROM data was collected at preoperative and postoperative assessment and measured active motion-arc of knee using an analogue goniometer. The goniometer had arm length of 31 cm and ROM was measured only once at each session during daytime without further specification with the patient supine in bed. Postoperative ROM was measured in clinical F/U at discharge and again at 3-month reassessment using the same method. All goniometer measurements were conducted by physiotherapists at our department, but we could not ensure all measurements were taken by the very same person throughout F/U period.

PROMs consisted of Oxford Knee Score (OKS) and the Forgotten Joint Score (FJS) recorded preoperatively at 3- and 12-months [18, 19] using electronic questionnaire.

Pain assessment after ~ 3 weeks (18–24 days) was collected using Visual Analogue Scale (VAS) described by the patients during activity and rest, respectively. Patients was requested to inform average feeling of pain from the operated knee at ~ 3 weeks F/U, without any further specifications (Table 2).

Knee circumference, which is a measurement of postoperative swelling, was measured postoperatively two times prior to discharge: 1) As soon as the patient was back from operation before starting rehabilitation and 2) immediately prior to discharge after meeting discharge criteria. Circumference measurements were as a consequence not timed to a specific time duration after operation. Knees in both instances were measured using flexible measuring tape placed around the leg orthogonal to leg axis via visual inspection. The tape was placed so it intersects the center of patella during full knee extension with patient supine. Measurement was done only once at each instance.

No approval from the National Ethics Committee was necessary as this was a non-interventional observational study, where we merely observed the consequences of a change in the usual rehabilitation regime offered to UKAs at our institution. Information was given and consent obtained from all patients orally during the pre-operative assessment and noted electronically in the local medical database. Permission to store and review patient data was obtained from the Danish Data Protection Agency Jr, No. 2007–58-0015. This research did not receive any specific grant from funding agencies in the public, commercial, or not-for-profit sectors.

### Statistics

Continuous data are presented as means with range, categorical data as absolute numbers and percentages (%). For comparison of variables between groups, student’s t-tests (unpaired T-test), paired T-test, Pearson’s chi-squared test (χ^2^-test), and Mann–Whitney U test were applied when appropriate. SPSS Statistics Software version 27.0 (IBM Corp.) was used along with R Studio version 1.3.1093 (RStudio, PBC). P-values less than 0.05 were considered statistically significant. Approaches to sample size justification for pilot and feasibility trials vary. We aimed for a target sample size of 50 patients as this would double the sample size recommendation of 12 participants per group for pilot studies put forward by Julious [20] as a rule of thumb. Data was assumed missing at random and no imputations were made.

## Results

**Fig. 2:**
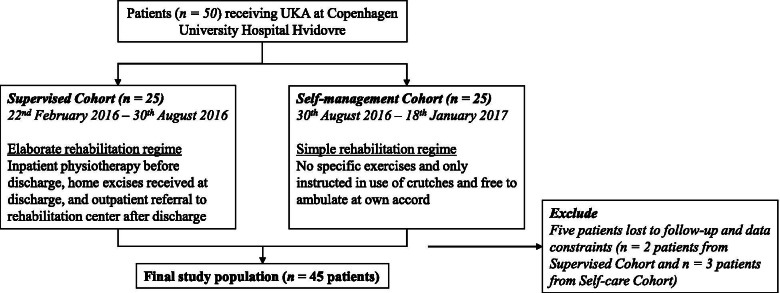
Patient selection process for Supervised and Self-management Cohort

Mortality during the F/U period was recorded with *n* = *1* patient dying during F/U in Self-management Cohort (at approximately 10 months F/U). This patient was included in analysis up until death. A total of *n* = *5* patients, two patients from Supervised Cohort and three patients from Self-management Cohort, were excluded due to missing PROM and ROM data (Fig. 2). No adverse events were recorded in the excluded patients. Complete data were obtained for *n* = *45* patients (Fig. 2). Patient demographics was similar between the two cohorts (Table 1

).

**Table 1:** Baseline characteristics

	*All patients*		
Variable	Supervised cohort	Self-management cohort	*p*
*Patients, n (%)*	23 (100)	22 (100)	
*Gender* *, * *Female/Male (% female)*	16/7 (69.6)	13/9 (59.1)	*0.46*
*Age (yrs), mean (range)*	66.6 (47–85)	65.3 (44–82)	*0.71*
*BMI, mean (SD)*	29.2 (4.93)	30.0 (6.50)	*0.62*
*Per-op complications, n (%)*	0 (0)	0 (0)	
*Pre-op total ROM, mean (SD)*	109.8 (9.94)	115.2 (14.01)	*0.14*
*Pre-op OKS, mean (95% CI)*	22.4 (6.76)	22.8 (6.78)	*0.85*
*Pre-op knee circumference, mean (95% CI)*	45.8 (3.69)	45.3 (4.67)	*0.70*

For normally distributed data, in accordance with Shapiro Wilk-test, kurtosis and skewness, mean values are presented, and *p*-value calculated using the unpaired T-test. *P*-values for categorical data is calculated using Pearson’s Chi-squared (χ2) test when appropriate

*Per-op* Peroperative, *Pre-op* Preoperative, *Per-op* Per-operative, *ROM* Range of Motion, *OKS* Oxford Knee Score, *SD* Standard deviation, *CI* Confidence interval

**Fig. 3:**
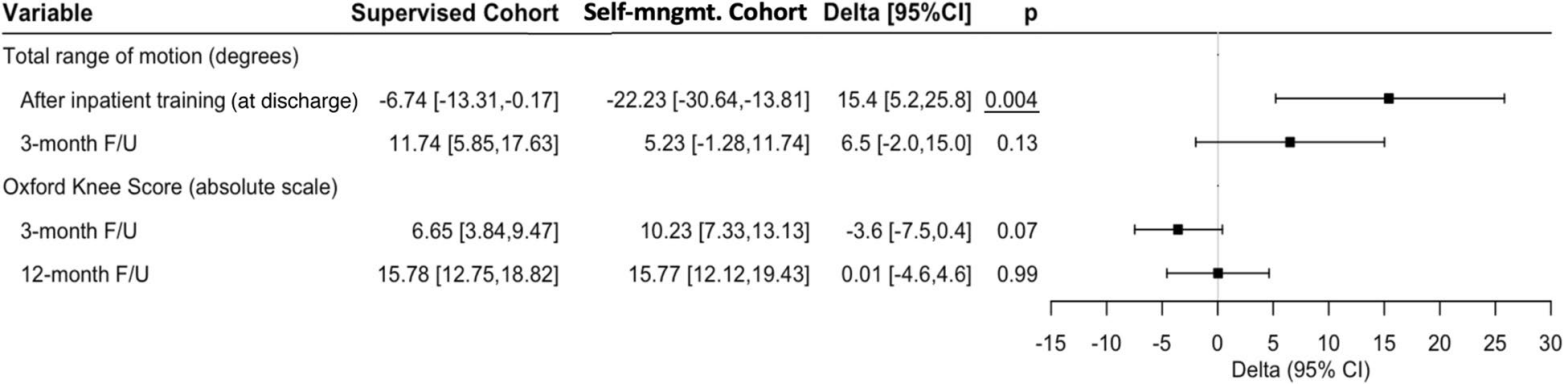
Between-cohort differences of ROM and OKS with respect to preoperative baseline. Normally distributed data, in accordance with Shapiro Wilk-test, kurtosis and skewness are calculated using unpaired T-test. Significant p values underlined. Units: ROM: Range of Motion; F/U: Follow-up; CI: Confidence Interval; Self-mngmt.: Self-management

Postoperative measurements were compared with their respective preoperative baseline for each cohort to find net difference (delta) used in comparison between cohorts. A significant difference of 15.4° (CI: 5.2°, 25.8°, *p* = *0.004*) in total knee joint ROM at discharge was seen between deltas in cohorts. Self-management Cohort showed a significant ROM reduction of -22.2° (CI: -30.6°, -13.8°) between preoperative assessment and discharge as opposed to Supervised Cohort -6.7° (CI: -13.3°, -0.2°). At 3-month F/U no significant between-cohort difference was observed in total knee joint ROM (Fig. 3).

In absolute figures, mean total ROM at discharge after initial inpatient rehabilitation was 103.0° for Supervised Cohort compared to 93.0° in Self-management Cohort (*p* = *0.015*). The relative smaller ROM in Self-management Cohort compared to Supervised Cohort was driven by a smaller relative range of flexion movement in knee joint of 11.7° (*p* = *0.009*). Later, at 3-month F/U no difference was seen in ROM between cohorts (*p* = *0.78*) (Table 2). OKS and FJS at 3- and 12-months showed no between-cohort differences nor absolute difference across cohorts (Table 2 and Fig. 3).

**Table 2:** Outcomes for supervised- and self-management cohort

	*All patients*		
Variable	Supervised cohort	Self-management cohort	*p*
*ROM at discharge*			
Extension, *mean (95% CI)*	5.4 (3.9–6.9)	7.1 (5.4–8.8)	*0.14*
Flexion, *mean (95% CI)*	97.6 (91.5–103.7)	85.9 (79.4–92.4)	*0.009*
Total range, *mean (95% CI)*	103.0 (97.8–108.3)	93.0 (86.7–99.3)	*0.015*
*ROM 3-month F/U*			
Extension, *median (range)*	0 (0–10)	0 (0–0)	
Flexion, *mean (95% CI)*	120.9 (113.9–127.9)	120.5 (116.1–124.8)	0.92
Total range, *mean (95% CI)*	121.5 (114.8–128.3)	120.5 (116.1–124.8)	0.78
*Oxford Knee Score*			
3-month F/U, *mean (95% CI)*	28.9 (25.0–32.8)	33.0 (30.4–35.5)	*0.07*
12-month F/U, *median (range)*	39.5 (19–48)	41.0 (16–47)	*0.47*
*Forgotten Joint Score*			
3-month F/U, *mean (95% CI)*	39.2 (31.5–47.0)	42.9 (33.2–52.5)	*0.54*
12-month F/U, *mean (95% CI)*	62.8 (52.0–73.6)	61.1 (49.3–73.0)	*0.83*
*Knee circumference*			
Before inpatient training, *mean (95% CI)*	47.3 (45.7–49.0)	46.4 (44.3–48.4)	*0.47*
After inpatient training, *mean (95% CI)*	47.3 (45.6–49.0)	46.7 (44.6–48.7)	*0.62*
VAS pain score 3-week F/U* (scale 0–100)			
Activity, *mean (95% CI)*	34.1 (23.8–44.5)	32.9 (25.5–40.4)	*0.84*
Rest, *median (range)*	17 (0–86)	12 (0–72)	*0.73*
*Length of Stay*			
*median (range)*	1 (0–4)	1 (0–2)	*0.67*
*Readmissions*			
< *30 days, n (%)*	1 (4.3)	0 (0.0)	*1*

For normally distributed data, in accordance with Shapiro Wilk-test, kurtosis and skewness, mean values are presented, and p-value calculated using the unpaired T-test, otherwise Man Whitney U test was applied. Significant *p* values are underlined

*ROM* Range of Motion, *Post-op* Postoperative, *F/U* Follow-up, *Active* during physical activity, *Passive* during no physical activity or stress, *CI* Confidence interval

^*^ *n* = *5 patients with missing data*

No significant differences were observed in LOS, readmissions < 30 d, VAS pain score during rest and activity at ~ 3 weeks F/U, and knee circumference before and after initial training (Table 2). No adverse events, harms, or complications including brisement to any patients were observed throughout this study.

## Discussion

In this pilot study of 45 consecutive unilateral UKA patients, we investigated and compared a range of knee outcomes in two cohorts. At discharge, after inpatient training, we found significant difference in total knee range of motion in Self-management Cohort compared to Supervised Cohort (*p* = 0.015) driven by difference in knee flexion (*p* = 0.009). This did not translate to detectable differences in any outcome including ROM (at 3-month F/U) and PROMs (at 3- and 12-month F/U) (Table 2). With respect to net differences in cohorts from preoperative baseline, no significant differences were witnessed between cohorts following UKA surgery apart from total knee joint ROM at discharge had significantly greater reduction in Self-management cohort (*p* = 0.004) (Fig. 3). No study to date present in the literature has investigated how two fundamentally different knee rehabilitation regimes affects UKA patients after UKA surgery.

The main limitations of this study are small number of included patients and study design. As this was a pilot study, no sample size analysis was performed. The study population is limited, and this naturally provides a substantial probability of risk for type-II error due to reciprocal relationship between effect size and size of study populations. This may hide real differences between cohorts. Also, our study only examined two prespecified regimes, which eliminates the option for a wider comparison between other strategies. However, this was also an advantage since the included rehabilitation regimes accounted for two extremes with respect to supervision and structure. Another reason was to limit confounding by focus on single center patients and thereby avoiding normal variations found in multi-center studies. Further on, knee ROM was measured only once during daytime without further specification, which may contribute to imprecision given natural variances in active motion arc during the day and after workouts. We did not measure preoperative daily activity levels in patients nor measure actual consumption of postoperative pain medication. Finally, we could not determine compliance to outpatient rehabilitation for Supervised Cohort, as data on actual attendance to outpatient municipality-based training sessions was unavailable.

We found significantly higher knee joint ROM at discharge in the Supervised cohort compared to the Self-management Cohort. As the Supervised Cohort partook in a range of physiotherapeutic exercises which include movements with full knee joint range (Fig. 1), it is not surprising we found improvement to ROM. However, the improvements in ROM diminished at 3 and 12 -months F/U, which suggests that early reductions found in ROM at discharge have negligible impact on risk of reduced ROM at longer term F/U for UKA patients. From TKA studies we know prognostic relevance of reduction in ROM at discharge has been found to be of very limited utility to predict later 6-month ROM and functional outcome [21], which concur with our findings. A better indicator for prognostic utility is possibly preoperative ROM and PROMs, as has been the case for TKAs [21], but since we found no significant difference in these preoperative variables, we are unable to explore this possible relationship in this pilot study.

**Fig. 4:**
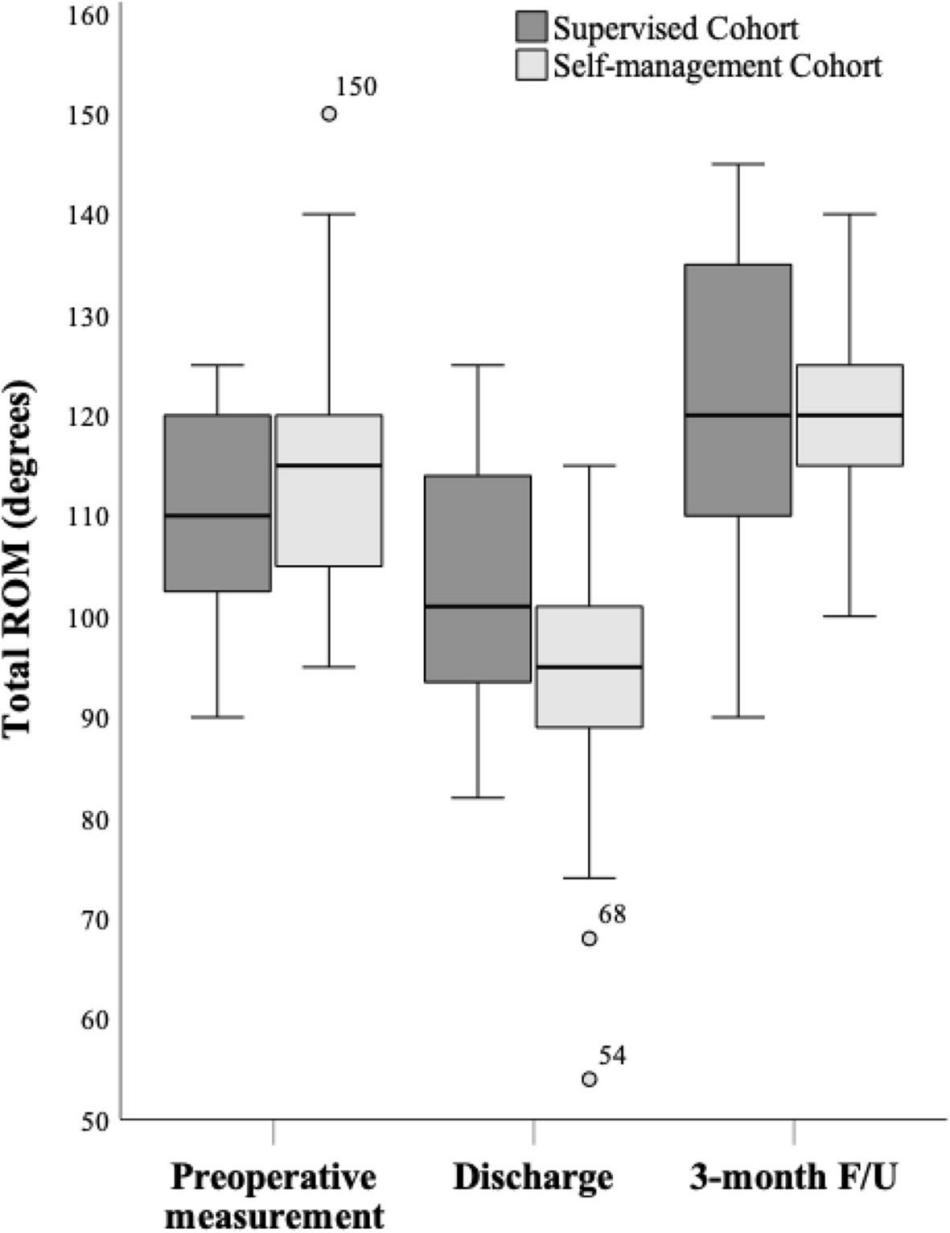
Total ROM at preoperative, discharge and 3-month follow-up. The boxes represent the interquartile (IQ) range. Median indicated by line across the boxes. The whiskers are no greater than 1.5 times the IQ range. Outliers are cases with values between 1.5 and 3 times the IQ range depicted by circles and labeled with specific ROM measured in degrees. ROM: Range of motion; Pre-op: Preoperative; Post-op: Postoperative measured at discharge; F/U: Follow-up

At 3-month F/U, only Supervised Cohort showed significant increase in total ROM at 3-months F/U (+ 11.7°, CI: 5.9;17.6, p < 0.001) while Self-management Cohort failed to do so (+ 5.2°, CI: -1.3;11.7, *p* = 0.11), when comparing between-cohort differences with respect to preoperative baseline (Fig. 3). A possible underlying cause for this dissimilarity can be partly attributed to Self-management Cohort may have had greater knee mobility at preoperative measurement of total ROM (115.2 vs. 109.8), and therefore Self-management Cohort indeed had less potential for later improvement following adaptation of identical UKA knee prosthesis (Fig. 2). Another observation is the safety aspect of the self-management regime, as no negative outliers were observed at 3-month F/U (Fig. 4). This is interesting as measurements at discharge training show two negative outliers in Self-management Cohort with total ROM of 54° and 68°, which is a reduction in ROM that is risks interference with activity of daily living [22]. Hence it seems all patients ultimately restore ROM before 3-month F/U regardless of regime and none experience horrific joint limitations.

With due precaution for study design our results may indicate outpatient physiotherapy has little effect on ROM beyond discharge following UKA. This was mirrored in reviews on TKA patients who received outpatient physiotherapy which showed no longer-term improvements to range of knee motion when compared to minimal physiotherapy or home-based rehabilitation [12, 23]. Also, Mahomed et al. previously showed group-based therapy is just as efficient as no or minimal physiotherapy in improving postoperative TKA outcomes. Furthermore, they showed patients appreciate the convenience of staying at home due to lack of traveling, as some patients have to rely on others for travel, due to typical temporary driving restrictions imposed on TKA patients [24]. Outpatient physiotherapy may have larger efficacy on knee patients experiencing perioperative complications [25], but as no complications was found for either cohort (Table 1) this remains speculative for UKA patients. When examining the literature for discharge criteria for knee patients, there is no univocal evidence in support of particular ROM targets before discharge [26, 27], and as few patients in general satisfy discharge criteria which incorporate any ROM threshold [28] functional discharge criteria are recommended for UKA patients [27], which resonate with our results.

In summary, we found gains in ROM following supervised rehabilitation are temporary with no significant difference in ROM at 3-months F/U nor PROM scores at 3- and 12-months F/U. This however needs verification in larger future studies.

No significant difference was found in OKS and FJS at 3- or 12-months F/U when cohorts were compared (Table 2). It seems neither inpatient physiotherapy during hospital stay nor outpatient referral to rehabilitation center provides a detectable discrepancy in OKS and FJS between cohorts. Also, worse ROM at discharge in Self-management Cohort seems to have no impact on PROMs at 3- and 12-months F/U. The limited nature of the study design and choice of PROMs might play a decisive role in these results.

For adequate comparison of LOS this study is too limited as median LOS for UKAs were found to be 1 day for both cohorts. It remains speculative if speed with which patients can meet functional discharge criteria vary when inpatient approach is changed. While other studies have discussed this [29, 30], it was out of scope for this study.

Albeit ensuring an optimal knee result and patient satisfaction after UKA is imperative, economic considerations in UKA rehabilitation are likewise key in a setting of rising health care costs and warrants us to consider identification of redundant aspects of patient care. Several other orthopeadic procedures sets president through their findings of indifference in outcome as some suggests removal of required formalized/supervised training for inpatient [31, 32] and outpatient physical therapy [33–35]. With UKAs rising popularity, it seems more important than ever to narrow in on the optimal rehabilitation approach and concurrently avoid inefficient rehabilitation efforts. Regarding the monetary costs, yearly healthcare costs following TKA are estimated to be 6-10kEUR higher compared to the reference population [36]. Although UKA patients have demonstrated slight cost reductions in comparison to TKA, they remain a costly patient population where even smaller improvements can lead to considerable socio-economic savings [37, 38]. In addition, prudent upstream preoperative assessment to ensure optimal selection for surgery is important. Yet, cost benefit analysis of physiotherapy, also as a viable alternative to operation, is out of scope for this study.

Development of new studies involving strict scrutiny of rehabilitation strategy for UKA patients is justifiably necessitated for both patient- and economic concerns, as these patients have been largely overlooked in the current literature [30, 39]. While UKA and TKA patients overlap considerably on several areas, it is crucial to remember UKA surgery is less invasive, cause less trauma, and has a separate set of indications and unique outcomes [8, 40, 41]. As such, rehabilitation efforts proven on TKA patients may not be directly transferable, as less invasive surgery might suffice with less supervision and larger degree of self-management. To elucidate this relationship, larger specific studies on UKA rehabilitation are desired.

In conclusion, transition to a simple rehabilitation regime following UKA surgery was associated with decreased ROM at discharge, which was not present at 3-months F/U. Interestingly, we found no other between-cohort differences for any other outcomes including OKS and FJS at 3- and 12-months F/U, although the study was likely underpowered for these outcomes. We encourage large-scale replication of these findings using randomized designs.

## Data Availability

The datasets generated and/or analysed during the current study are not publicly available due to compromised patient privacy but are available from the corresponding author on reasonable request.
